# Changing Children’s Understanding of the Brain: A Longitudinal Study of the Royal Institution Christmas Lectures as a Measure of Public Engagement

**DOI:** 10.1371/journal.pone.0080928

**Published:** 2013-11-15

**Authors:** Nathalia L. Gjersoe, Bruce Hood

**Affiliations:** 1 Childhood & Youth Study Centre, Open University, Milton Keynes, Buckinghamshire, United Kingdom; 2 Bristol Cognitive Development Centre, Bristol University, Bristol, Avon, United Kingdom; The University of Western Ontario, Canada

## Abstract

Demonstrating the impact of public engagement is an increasingly important activity for today’s academics and researchers. The difficulty is that many areas of interest do not lend themselves well to evaluation because the impact of each single intervention can be hard to trace and take time to become manifest. With this in mind, we evaluated a lecture based around the 2011 Royal Institution Christmas Lectures, ”Meet Your Brain,” delivered to school children from low performing schools. We compared knowledge about four neuroscience facts one week before, one week after and six weeks after the lecture. Analysis revealed significant knowledge transfer one week after the lecture that was retained five weeks later. We conclude that public engagement through tailored lectures can have significant impact in the moderate term with the potential to leave a lasting impression over a longer period.

## Introduction

In recent years, the terms “engagement” and “impact” have become familiar amongst scientists, universities and research centers that receive financial support from the public. Increasingly, government funding agencies have recognized that it is vitally important to engage the nation with publicly funded science in meaningful ways [1-3]. For example, in 2010, the Concordat for Engaging the Public with Research was signed by the UK research councils that “recognizes the importance of public engagement to help maximize the social and economic impact of UK research.”[4] The Concordat is designed to support activities that include working with science centers, creating opportunities to inform research questions, presenting to the public, engaging with young people and contributing to new media.

These objectives are to be evaluated and implemented in a nation-wide analysis of research activities that will have major financial implications for the UK’s Higher Education Institutes (HEIs). In 2014, the UK Research Excellence Framework (REF) will seek to provide a basis for resource allocation, accountability for public investment in research, benchmarking information and reputational yardsticks [5]. For the first time, this exercise will require HEI’s to submit a statement about their approach to, and strategy for, enabling research impact, supported by a set of case studies containing “a narrative that includes indicators and evidence as appropriate to the case being made.”[6] Research impact here is targeted at non-academic effects, changes or benefits to society and will account for 20% of the overall assessment outcomes, alongside the quality of research outputs (65%) and the vitality of the research environment (15%) [6].

Already researchers have had to consider impact as UK government funded grant proposals require that applicants lay out a detailed plan of public engagement and pathways to impact. Substantial efforts have been made to create usable models of impact for public engagement with science (e.g. Rowe & Fewer, 2005 [7]; British Science Association, 2010 [8]; Trench, 2008 [9]) but the field remains divided. While there is general recognition of the importance of measuring impact, there is little agreement about what public engagement ‘is’, what to measure or who of the engaged parties to evaluate (Wellcome Trust, 2012 [10]). Moreover, evaluating impact on public opinion, knowledge or activity is very difficult because of the myriad of stimuli and interventions the public are subject to. Rarely is it possible to trace an opinion or a course of action down to one particular intervention. Impacts are easily quantifiable when one is dealing with technologies and applications that provide objective data such as innovations, sales or quantitative changes in practices or procedures, but less measurable outcomes such as knowledge transfer or education of the general public is much more problematic outside of a formal examination environment [11].

Many academics engage with the public through lectures and events such as science festivals but, despite extensive efforts, defining and measuring effects remains a somewhat elusive task. An important distinction to arise from research in this area is that it is not enough to show that dissemination has been extensive (‘reach’) without also showing that there has been lasting effect of exposure on thoughts and behaviour (‘significance’) [12]. The current challenge is for proponents of public engagement to demonstrate through statistical methodology and analysis that these activities achieve objectively valid and reliable measures of impact as defined by reach and significance.

One public engagement event that has been popular for well over a hundred years in the UK is the Royal Institution (Ri) Christmas Lectures [13]. These lectures, instigated by Michael Faraday in1825, are aimed at a teenage audience and since 1966 have been broadcast on national television. They are generally considered the pinnacle of scientific public engagement and have been given by notable figures in public engagement including Sir David Attenborough, Richard Dawkins and Carl Sagan. Even though viewing figures attest to the popularity of these lectures, demonstrating considerable reach, there has been no experimental measure of their significant lasting effect.

In 2011, the second author was invited to present the Christmas Lectures entitled, “Meet Your Brain.” The lectures were divided into three themes, “What’s inside your head?” (introduction to basic neural architecture), “Who’s in control anyway? (executive control including memory, planning, inhibition and attention) and “Are you thinking what I’m thinking?” (social brain mechanisms). Overall viewing figures were estimated for the first broadcast to be 1.06 million with an audience appreciation index average of 89% based on independent polling analysis (personal communication from the BBC to second author). Any figure over 85% is rated as excellent. These figures are evidence for reach as well as popularity but is there any evidence that there is also impact of the content communicated in terms of significant lasting effect beyond entertainment? 

To address that question, we designed a study to test the moderate long term impact of segments taken from the “Meet Your Brain” in a longitudinal, repeated measures study of school-children’s retention of knowledge of information delivered in the Christmas Lectures. We selected a group of Bristol schoolchildren for a number of practical, methodological and validity issues. First, it was impractical to assess the original children’s audience of around 900 individuals, as the allocation of tickets to the lectures was beyond our control and each audience member only watched one of the three lectures. Second, we wanted to collect data in standardized circumstances that would test children’s knowledge in a relatively controlled environment where they could not look up answers or ask others for help. Third, the audience members of the original lectures in London were already motivated to learn about science and so it was judged more appropriate and valid to conduct our study on children from underperforming schools where there was greater need to demonstrate the value of public engagement for science education. 

## Methods

### Participants

250 students from form classes of 8 widening participation schools around Bristol were invited to attend the lecture. Widening participating schools were defined as those who scored in the lowest 40% of all schools and colleges on average score per A-Level entrant and percentage of students applying to Higher Education. The students were from a range of ethnic backgrounds. For analysis, students were divided into those from primary schools (N = 84, age range = 9-11, mean age = 10.6 years, 43 boys) and those from secondary schools (N = 85, age range = 12-14 years, mean age = 13.0 years, 43 boys). 

### Ethics Statement

Written informed consent was obtained from each school and the parents of each child who took part. Full ethical approval for the study was received from the Bristol University Faculty of Science Ethics Committee in accordance with the guidelines set out by the British Psychological Society. All responses were transcribed and stored anonymously on a secure server. 

### Materials

Three surveys were developed to explore the students’ understanding of specific psychological issues before and after the lecture. The validity of all questions and their format was assessed independently by two experienced science engagement professionals to ensure that the range and diversity of questions was appropriate for the age of students tested and sufficiently interesting to keep their attention. The surveys differed at each time-point to mediate against responders simply remembering what response they had given previously. To do this, distractor questions were included which are described below. Four primary questions of interest were repeated in every survey, enabling a repeated measure analysis of understanding and retention. These questions were chosen because surveys of 246 members of the audience of the Royal Institution Christmas Lectures before the lectures commenced revealed that these were common areas of misconception. The content of the intervention that the following results are based on was selected from the demonstrations used in the Christmas Lectures that delivered specific teaching for the 4 target questions. These were as follows:

Question 1. What are the building blocks of the brain? 

 a) muscles b) neurons c) lobes d) tendons

The structure of neurons was described by the lectures demonstrated with an illustration of a neuron accompanied with an animation of a nerve impulse propagating across a network of neurons, axons and synapses. The video used is reproduced here with permission from its creators: http://www.youtube.com/watch?v=-SHBnExxub8 [14].

Question 2. How fast do messages pass from brain to body? 

 a) instantly b) 1m per sec c) 5m per sec d) 10m per sec 

This was demonstrated with audience participation where 10 students in a row were asked to place one hand on their left-sided neighbour’s shoulder and squeeze it when they felt their other shoulder squeezed by the neighbour on the right. The lecturer began the demonstration by squeezing the first student’s shoulder and starting a timer that was stopped when the tenth student at the end shouted ‘Stop’. The demonstration was then repeated except each student now held their neighbours hand (rather than shoulder) thereby adding ten arm’s lengths to the overall distance, which is approximately an extra 10 metres. By subtracting the first reaction time from the second and dividing by ten, the lecturer was able to demonstrate that the speed of the neural transmission was approximately 10 metres per second. The lecturer added that this was simply an illustrative case because different parts of the body had different conduction times depending on the nerves that supplied them. A video of this demonstration as shown at the Ri Christmas Lectures is available here for illustration: http://www.youtube.com/watch?v=cn1qVZF-bI4 [15].

Question 3. We only use 10% of our brain? 

 a) True b) False c) don’t know

This was demonstrated by asking audience members to behave like individual neurons connected together by light ropes which represented the neural pathways between different sensory processing areas for shape, colour and taste. They were then asked to register different features of a particular fruit when presented. The demonstration revealed the parallel, distributed nature of representations, the effects of repeated stimulation on enhancing neuronal activity and how Hebbian learning occurs. It also emphasized Hebb’s two neurophysiological principles of “use it or lose it” and neurons that “fire together, wire together.” This point was emphasised to students – that it was impossible that they only used 10% of their brain because the rest would simply wither away [16]. A video of this demonstration as shown at the Ri Christmas Lectures is shown here for illustration: http://www.youtube.com/watch?v=mH2esaY1mvk [17].

Question 4. How are memories formed?

a) by association – things that happen together are automatically linked together

b) by training – deliberately making the brain link things together

c) like a flashbulb – some things are immediately imprinted on the memory

d) all of the above

This learning point was covered by several demonstrations including the Hebbian neural network example for question 3. We also used a mock robbery where a thief ran on to the stage to steal a teddy bear. Later, audience members were asked to solve the crime in an identification game demonstrating the unreliable nature of witness memory [18]. This was further emphasized in another demonstration using the Deese-Roediger-McDermott (DRM) paradigm [19]. The entire audience was asked to memorize a list of words read out to them by the lecturer. One minute later, they were asked whether or not target words were in the original list, again read out by the lecturer. One of the target words was semantically related to all of the words but was not present in the original list. Almost all of the schoolchildren falsely recognised the new target word as being present in the original list. The demonstrations revealed the reconstructed nature of memory and the effects of semantic relatedness, which can be explained by activation of representations that share the same neural networks in memory. Finally individual audience members were asked about the most memorable event that had happened to them so that the lecturer could discuss the concepts of autobiographical and “flashbulb” memories [20]. A detailed description of this demonstration, with stimuli, is given at a dedicated website we created to support the Christmas Lectures. This was launched after the current study was complete. The Deese-Roediger-McDermott (DRM) paradigm is described in the final section entitled ‘Memory Illusion’: http://thebrainbank.org.uk/teaching-materials/brain-function/memory/ [21]. 

#### Distractor Questions

In addition, a number of other demonstrations from the Ri Christmas Lectures were included based on those that a post-lecture survey of 137 audience members had said that they had found the most surprising or interesting and limited to those that we were able to reproduce in the new location. 

Distractor questions were different from one survey to the other and related to other demonstrations that had been shown in the lecture such as ‘Why is doing two activities at once sometimes difficult?’ and ‘Why do we have a brain?’ 

#### Interest questions

In addition, in Survey 2 (administered 1 week after the lecture) respondents were asked which of the demonstrations they found the most interesting, surprising and the least interesting and what question they would ask a brain scientist if they had the opportunity. In Survey 3 (administered 6 weeks after the lecture) they were asked whether there was any one demonstration that they remembered best from the lecture. This data was subsequently used to select a series of 60 activities and demonstrations that were included in a free online resource of teaching materials and notes which can be found here: www.thebrainbank.org.uk [21]. 

### Procedure

Schools from a widening participation database were invited to come to the university to take part in a 1-hour expert lecture about how brains worked. Schools were accepted on a first come-first served basis until the maximum capacity of the lecture theatre was filled (270 students and teachers). Only those who had received consent from their parents to attend the lecture and complete the surveys attended. Parents were given the option of their child attending without completing the surveys but no parents opted for this. The lecture was delivered live by the second author and all of the children saw the lecture at the same time, under the same conditions. Surveys were sent out one week before the lecture (Time 1), one week after the lecture (Time 2) and six weeks after the lecture (Time 3). Teachers distributed the surveys during class to ensure that they were all completed at the same time and the surveys were all collected on the same day to ensure the same amount of time had elapsed for all respondents. Each class received a £25 book voucher to thank them for their participation. 

### Analysis

Responses to the interest questions in Survey 2 (What did you find the most interesting? What did you find the most surprising? What did you find the least interesting?) were received from106 respondents. Overall, the most interesting and surprising demonstrations were the illusions (72%) and the most common response (43%) was that they found ‘nothing’ the least interesting. The rest of the responses were spread out relatively evenly across the different demonstrations. We received 69 responses to the memory question in Survey 3 (What do you remember best from the lecture?), the majority of which (75%) reported that they remembered the illusions best (demonstrations of the illusions can be viewed at http://www.thebrainbank.org.uk [21]). 

**Figure 1:**
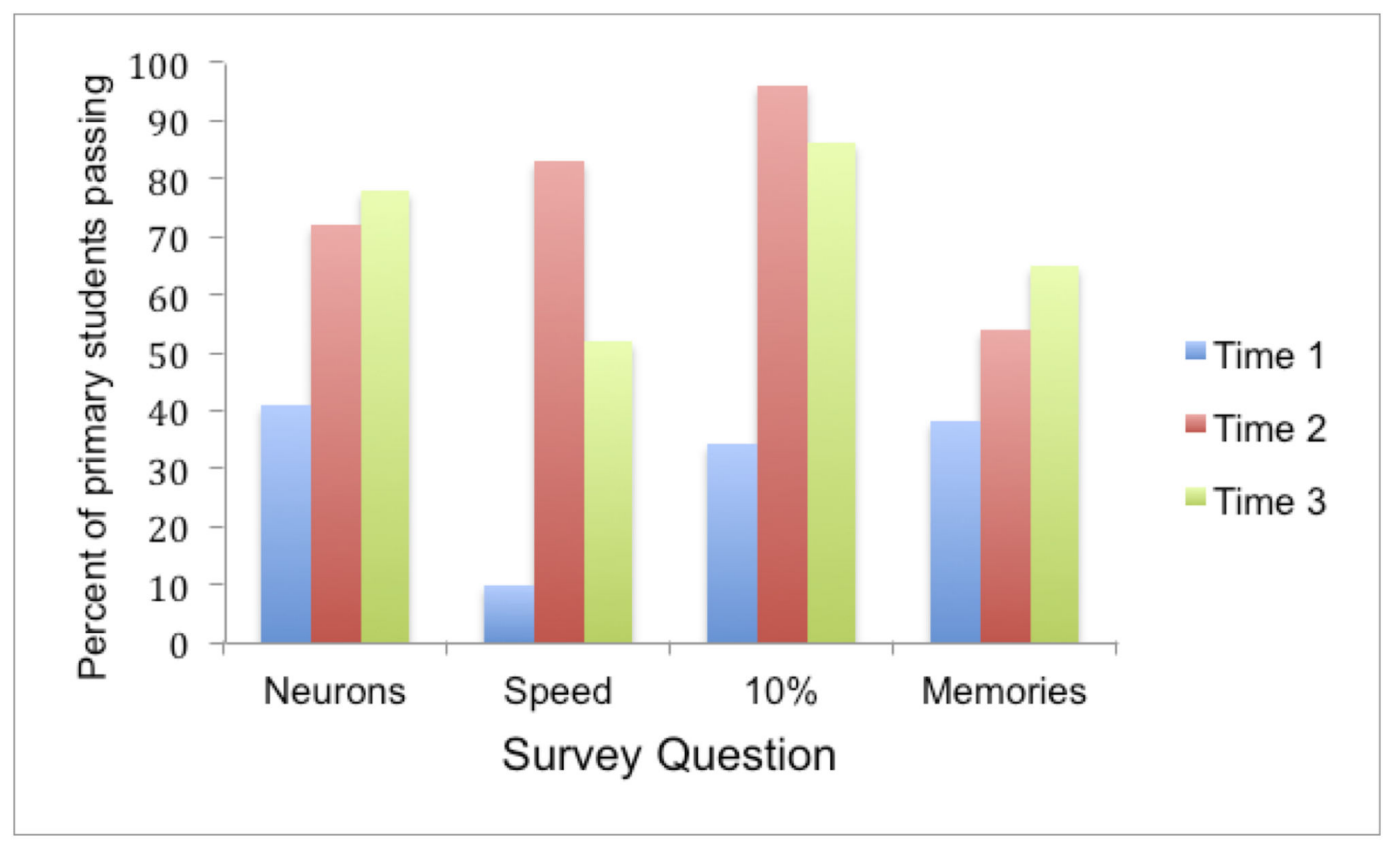
Percentage of correct responses from primary students for each question for each survey. Neurons refers to Question 1, Speed to Question 2, 10% to Question 3 and Memories to Question 4. The blue bars represent the percentage of primary school children passing each of the questions before the lecture. The red bars represent pass-rates 1-week after the lecture and the green bars represent pass-rates 6 weeks after the lecture. Chance pass-rates are 25% for questions 1, 2 and 4 and 33% for question 3. Significant or approaching significant increases in pass rates occurred on all questions between Times 1 and 2 and between Times 1 and 3.

**Figure 2:**
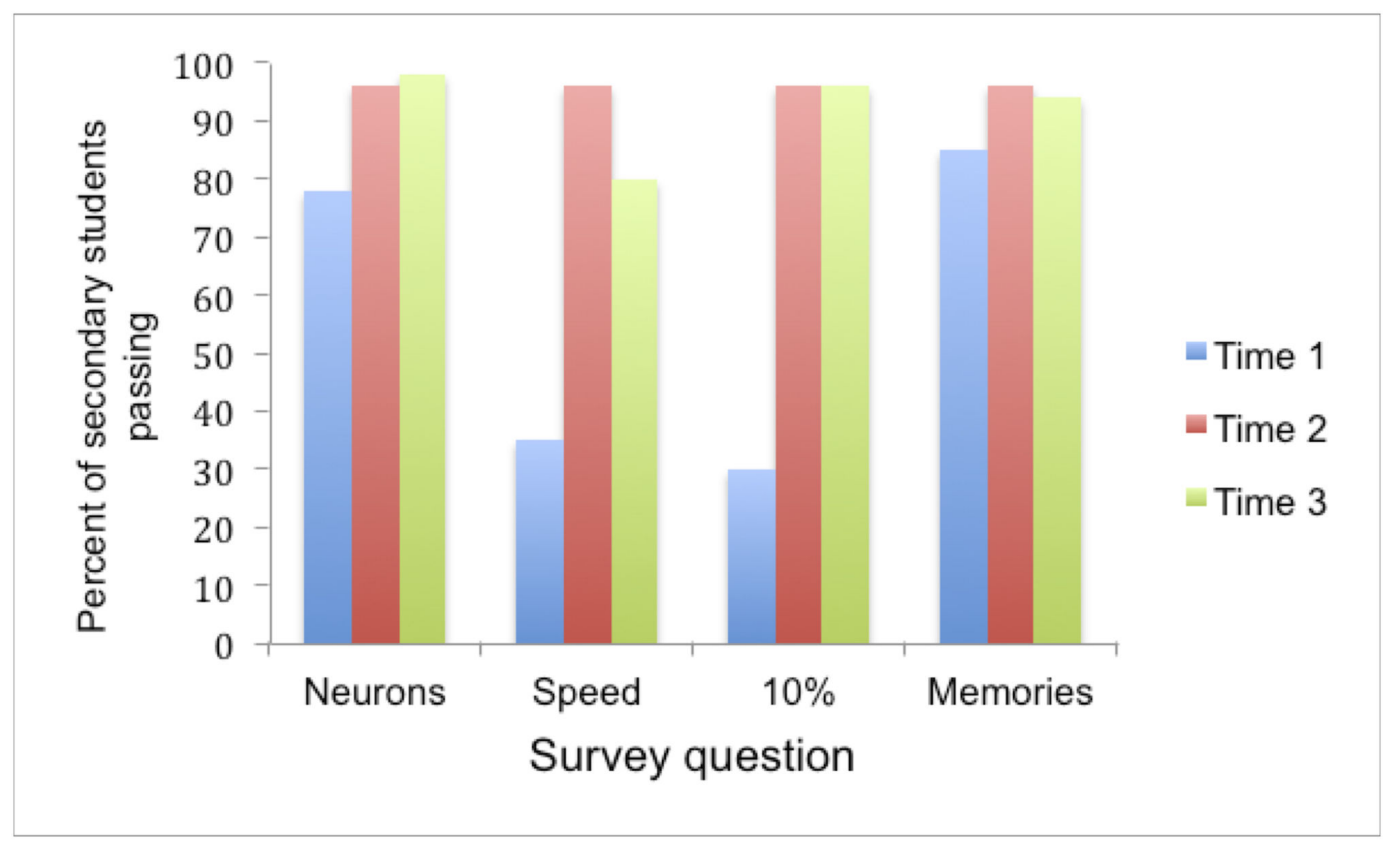
Percentage of responses from secondary students for each question for each survey. Neurons refers to Question 1, Speed to Question 2, 10% to Question 3 and Memories to Question 4. The blue bars represent the percentage of secondary school children passing each of the questions before the lecture, the red bars represent percentage pass-rates 1-week after the lecture and the green bars represent percentage pass-rates 6 weeks after the lecture. Chance pass-rates are 25% for questions 1, 2 and 4 and 33% for question 3. Significant increases in pass rates occurred on all questions between Times 1 and 2 and between Times 1 and 3.

Survey data for the test questions was collected from 232 children at Time 1, which represents 93% of the invited sample. Of this group, 169 returned surveys at Time 2, which represents 73% of the Time 1 sample. Finally, 117 returned a survey at Time 3, which represents 69% of the Time 2 sample. As all responses were coded into binary pass or fail, non-parametric statistics were used for all of the following analyses and bonferroni corrections applied. The mean group percentages of correct answers for each survey are shown in Figures 1 and 2. 

#### Baseline (Time 1)

There was no significant difference in pass-rates between the different primary schools or between the different secondary schools so data were collapsed within these groups (primary and secondary) in all subsequent analyses. Simple binomial analysis of answers at Time 1 revealed that before the lecture, as a group, the children did not know the correct answer for questions 2 and 3 but performance on question 1 (both primary and secondary) and question 4 (secondary but not primary) was surprisingly high even at baseline. This may have been because the audience had some familiarity already with neurons and secondary students had some familiarity with memory formation and may have covered these briefly in class. Significantly more secondary students passed questions 1, 2, and 4 at baseline than primary students (Question 1: Z = -4.1, p < 0.001, Question 2: Z = -4.43, p < 0.001; Question 4: Z = -5.79, p < 0.001) but this was not the case for Question 3 (Z = -5.79, p = 0.83). 

#### Times 1 and 2

To test whether there had been a significant change in responses one week following the lecture, a repeated measures non-parametric McNemar change test was applied to those surveys returned at Times 1 and 2. This analysis revealed there were significant increases in the proportion of correct answers on all questions (*Primary* - Question 1: χ^2^(84)=12.8, p < 0.001, Question 2: χ^2^(84)=59.16, p<0.001, Question 3: χ^2^(84)=44.46, p < 0.001; *Secondary* – Question 1: χ^2^(85) p < 0.01, Question 2: χ^2^(85) = 38.0, p < 0.001, Question 3: χ^2^(85) = 53.02, p < 0.001. The only exception was Question 4 to which the primary cohort’s response to Question 4 which was approaching significance, Primary - Question 4: χ^2^(84)=3.18, p = 0.07 and the secondary cohort’s response was not significant, p = 0.18. Overall there was no significant difference between primary and secondary students in the rate of improvement (pass-rates at Time 2 minus pass-rates at Time 1), Z=-1.48, p=0.14. 

#### Times 1, 2, and 3

An analysis was performed to determine if there was any further change on the proportion of correct and incorrect answers given five weeks later using the binomial distribution test on those children who provided data at all three time points. This population consisted of 71 primary students and 46 secondary students. This revealed that there was no significant change in pass-rates on target questions 1, 3, and 4. However, for Question 2 there were significantly fewer correct answers at Time 3 compared to Time 2, Primary: p < 0.001 (Mean pass-rate at Time 2 = 0.83 and Time 3 = 0.52); Secondary: p < 0.05 (Mean pass-rate at Time 2 = 0.96 and Time 3 = 0.80). 

However, final McNemar change tests revealed that correct responding was still better at Time 3 than that observed at Time 1, for all questions including Question 2, Primary – Question 1: χ^2^(71))=22.32, p < 0.001, Question 2: χ^2^(71)=26.28, p<0.001, Question 3: χ^2^(71)=30.14, p<0.001, Question 4: χ^2^(71)=11.17, p<0.01; Secondary – Question 1: p <0.02, Question 2: χ^2^(46) = 14.82, p<0.001, Question 3: χ^2^(46)=30.03, p<0.001. The one exception was the secondary cohort’s responses to Question 4 which were consistently high across all the surveys and there was no significant difference between pass-rates at Time 1 and Time 3: Question 4: p = 0.289. Overall there was no significant difference between primary and secondary students in the rate of improvement between Time 1 and Time 3 (Z=-0.09, p=0.93). 

## Discussion

Despite the emphasis on public engagement and impact from government funded grant agencies and the general consensus that it is important for the good of society to make these activities a core component of academic and research activities, it has proven difficult to conduct long-term follow-up statistical studies to evaluate the reach and significance of these efforts due to the fact that they are often one-off events with little subsequent access to the audience. In this study, we presented schoolchildren with a lecture about basic neuroscience designed around the content of the 2011 Royal Institution Christmas Lectures and evaluated knowledge one week before, one week after and six weeks after the lecture. Repeated measures analysis enabled us to determine whether there had been a significant lasting retention of understanding as evidenced by recognition memory. The general finding was that prior to the lecture there was ignorance concerning the content to be delivered in the lecture, but a significant educational shift one week after the lecture with approximately four out of every 5 children recognizing the correct answer to questions. This impact was significant because children also remembered the correct answers six weeks after the lecture. Although significantly more secondary than primary students passed the test questions at baseline, no difference was found in the degree of improvement or retention from Time 1 to Times 2 and 3 between these two age groups. In terms of long-term impact this is significant because a meta-analysis of knowledge retention in schoolchildren [22] has concluded that the most significant decline in recognition memory occurs between one week and six weeks and then levels off, indicating that our lecture probably left a lasting impression. 

However not all content areas were equivalent and not all retention faired equally well after six weeks. Remembering the speed of neural transmission in the example given (Question 2) tended to be remembered less well but this may have been a consequence of the structure of the answers both in the number and the similarity of the alternatives. However, it was still answered correctly by more children six weeks after the lecture than one week before. One remarkable finding was that a common misconception about the brain, namely that we only use 10%, was widely endorsed by children before the lecture but consistently rejected at both Times 2 and 3. This is particularly important given the prevalence of this belief in popular culture [23]. Indeed the idea that we only use 10% of our brain is so pervasive that 30% of US psychology university students [24] and 59% of university educated Brazilian adults have been reported to agree with this claim [25]. It is a misconception that forms the basis of many lucrative practices and pseudoscientific claims about improving or enhancing our brain potential and is frequently used in advertising campaigns [26]. Our study demonstrates that public engagement with schoolchildren can combat this significant public misconception but it remains an open question whether they eventually succumb once again to this cultural myth. 

While we are encouraged by this initial study, some caveats need to be considered. Compliance for our study was initially good but there was declining participation with time, so that only around 50% of the children provided all three sets of data. It is possible that our significant findings could be biased by sampling errors of those who failed to return the surveys during the requested time slot. These children may not have shown the patterns of retention we report here. Various reasons were given by the schools but mostly, they concerned scheduling issues and other competing priorities placed upon the teachers. However, it is worth noting that it was the teachers and/or school that were unable to respond rather than individual children and comparison at baseline revealed no significant difference in response pattern between schools so we have no reason to believe that the missing data represent a different type of child who would not have retained the knowledge. 

It is also unclear from this study what impact on memory the repeated questioning may have had. For instance, the demonstrations illustrating the correct response to the test questions may have been especially well remembered simply because the students were repeatedly asked about them. To address this issue, it may be worthwhile repeating the study but surveying only half of the members of the audience at Time 1 and Time 2 and then comparing responses at Time 3 between those who have been asked the same questions repeatedly and those that have not. However, such a manipulation was beyond the scope of the current study. It is also interesting to note, counter to this potential criticism, that when asked specifically which demonstrations were remembered best at Time 3, none of the students listed any of the demonstrations relevant to the test questions. Neither did students receive any feedback regarding correct responses at Time 2 upon which to base their answers at Time 3. 

Another possibility is that some teachers may have reinforced the learning points in class after the lectures. However, surveys of the teachers asking whether they thought they would use any of the activities in class revealed that all of the teachers saw the main benefits of the lecture as being with regards to general student interest and having experience of being in the university rather than seeing specifically relevant links to the curriculum. As such, they did not think they would use the contents of the lecture directly in class so it seems unlikely that some students received unreported re-enforcement between surveys. 

There are also other measures of impact that we did not evaluate that are arguably more important such as a positive change in attitudes towards science and neuroscience in particular. However, a report [27] from the UK National Centre for Coordinating Public Engagement has recently identified that one of the key challenges for evaluating the impact of public engagement is to move beyond advocacy based on single case studies towards “rigorous, robust studies that are able to withstand sustained scrutiny.” We believe that our study satisfies this recommendation by demonstrating an achievable evaluation technique that is statistically significant. That said, there are considerable difficulties undertaking the longitudinal evaluation we applied as evidenced by the drop-out and there are major cost and resource implications. However, our findings indicate that public lectures can deliver content that schoolchildren are likely to remember months afterwards. 
